# Current and future uses of genetic improvement technologies in livestock breeding programs

**DOI:** 10.1093/af/vfae042

**Published:** 2025-04-05

**Authors:** Alison L Van Eenennaam

**Affiliations:** Department of Animal Science, University of California, Davis, CA 95616, USA

**Keywords:** artificial insemination, cattle, genetic improvement, gene editing, genomic selection

**Implications**

• Genetic improvement has been a key contributor to the sustainability of animal agriculture, but these changes in overall production and efficiency have not been universal, and many low- and middle-income countries (**LMIC**) have production systems with high emission intensities.
• The adoption of cost-effective, genetic, feed, and nutrition practices, and improving livestock health in LMIC are seen as one of the most promising interventions to reduce emissions resulting from projected increased demand for animal-source food through 2050.
• Genetic improvement has a proven track record of productivity enhancements, and following implementation, genetic improvement is permanent and cumulative.
• Gene editing offers an approach to introduce useful genetic variation into future cattle breeding programs in the absence of the linkage drag that typically accompanies traditional introgression of useful alleles through crossbreeding, although its adoption will likely depend on whether global regulatory approaches facilitate public acceptance and free trade of milk, meat, and germplasm derived from GnEd animals.
• Delaying access to genetic improvement technologies to tackle otherwise intractable problems like animal disease, as happened with genetic engineering, is associated with a high opportunity cost of unrealized benefits.

## Introduction

Genetic improvement of food-producing species is a powerful tool for improving the sustainability of animal agriculture. Conventional selection programs, beginning with selective breeding using statistical prediction methods, such as estimated breeding values and more recently genomic selection, in synergistic combination with reproductive technologies (e.g., artificial insemination), have accelerated the rate of genetic gain by enabling more accurate selection and intense utilization of genetically superior parents for the next generation. The aims of this article were to review the current uses and opportunities to use existing genetic improvement technologies to improve milk and beef production efficiencies, with an emphasis on the large cattle populations in low- and middle-income countries (**LMIC**). Then, future opportunities that might arise from gene editing (**GnEd**) are considered, with some focus on the regulatory status of extant GnEd food animal applications and public perception. For GnEd to be adopted, it must be able to scale and integrate smoothly into the operation of existing cattle genetic improvement schemes. Finally, the opportunity cost of inaction and delays in the adoption of genetic improvement technologies is discussed in the context of animal breeding.

## Global Cattle Populations

**Figure 1.**
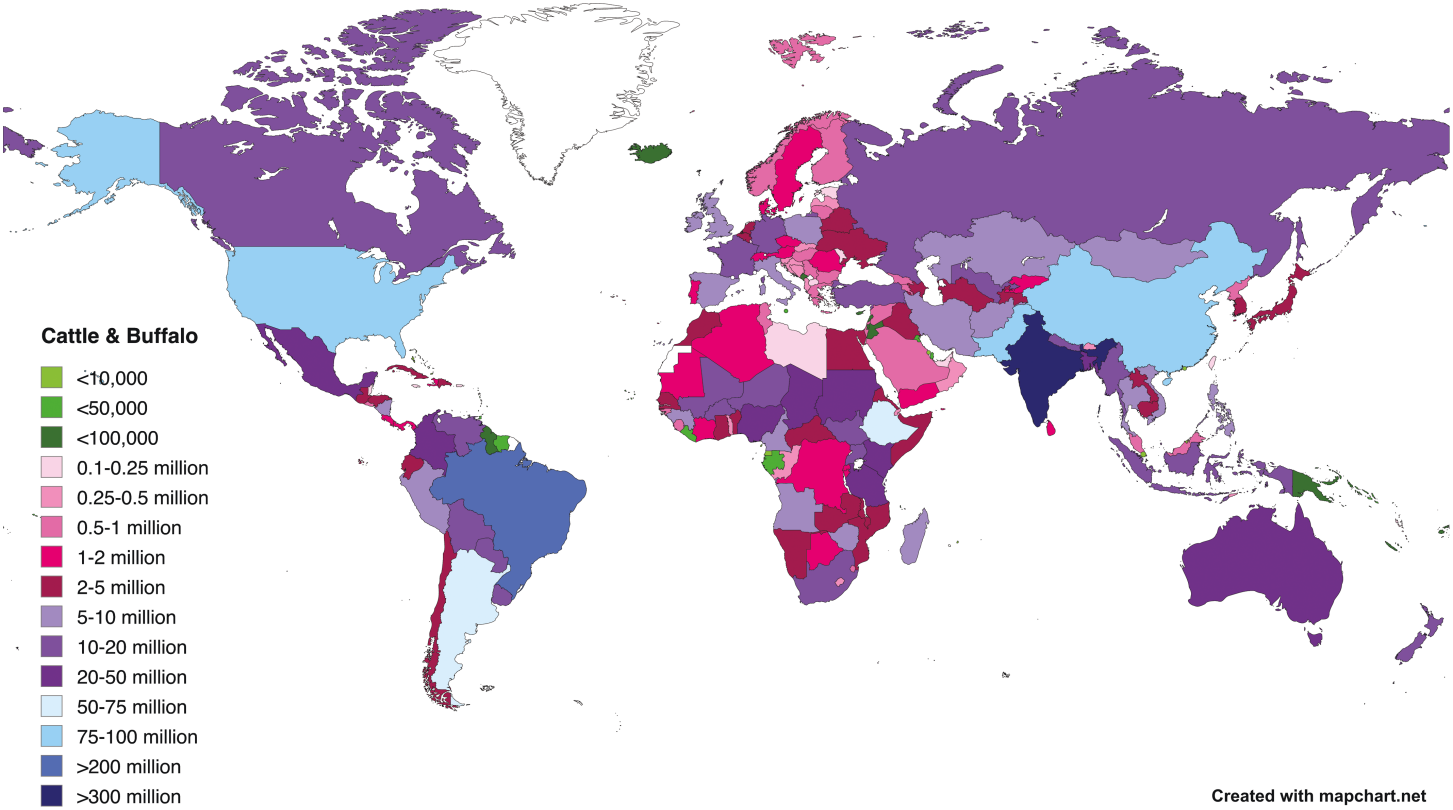
Cattle and buffalo numbers by country 2022 (FAO, 2024).

In 2022, the global cattle (155.2 billion) and buffalo (205.1 million) population (Figure 1) collectively produced 76.3 MMT of bovine meat and 753 and 144 MMT of cow and buffalo milk, respectively. The United States produced 18.6% (12.9 MMT) of the world’s beef with 6% (92.1 million) of the world’s cattle in 2022. Brazil with 15% (225 million) of the world’s cattle population produced 15% (10.4 MMT) of the beef, whereas India with 194 million cattle and 112 million buffalo produced only 5.8% (4.35 MMT) of bovine meat. Similarly, the African continent with almost 25% of the world’s cattle produced around 9.0% beef. There are a myriad of reasons for these differences including the fact that cattle are also raised for milk, hides, manure, and draught power and that cattle serve religious and other roles not captured by the single metric of beef output.

**Figure 2.**
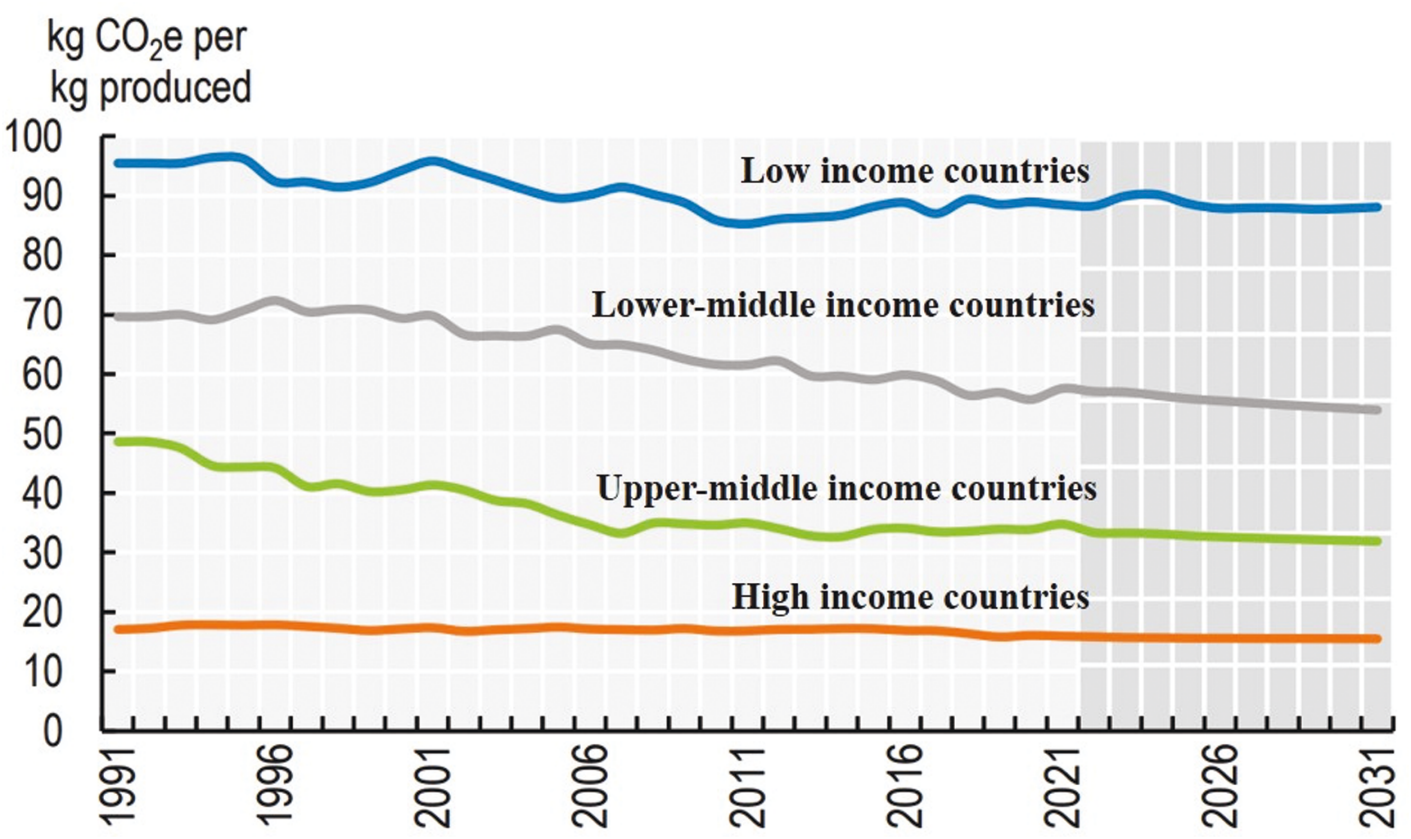
GHG emissions intensity per kg beef produced per region through 2031. OECD calculations based on FAOSTAT-Emissions Totals, Statistical Division of the Food and Agriculture Organization of the United Nations (FAO), Rome (OECD and FAO, 2022).

Where and how cattle are raised has important implications on the efficiency and greenhouse gas (**GHG**) emissions intensity (emissions produced per unit of product) of beef and milk production. Many LMICs have production systems with high emission intensities (Figure 2), and this is important because LMICs are home to 76% of the global cattle herd and contribute 75% of the global ruminant GHG emissions. The adoption of cost-effective, genetic, feed, and nutrition practices, and improving livestock health in LMIC are seen as the most promising interventions to reduce emissions resulting from projected increased terrestrial animal-source food demand through 2050 (FAO, 2023). It has been estimated that as compared to a baseline where emission intensities are held constant in the future, improving livestock production efficiencies in the 10 countries with the largest emission reduction potential (Madagascar, Morocco, Niger, South Africa, Tanzania), Asia (China, India, Iran, Turkey) and South America (Brazil) could contribute 60% to 65% of the global reduction in livestock emissions by 2050 (Chang et al., 2021).

Milk constitutes 67% of the total protein produced by cattle. India, the United States of America, and China are the top three countries in terms of raw milk production from cattle, with the European Union as a region being second only to India. Brazil is the fourth largest milk producer, despite having the world’s largest cattle herd. World milk production is projected to grow at 1.5% p.a. over the next decade to 1,039 MMT in 2032, faster than most other main agricultural commodities. Over half of the increase in total milk production is anticipated to come from India and Pakistan, which will jointly account for over 32% of world production by 2032. According to a 2022 OECD report (OECD and FAO, 2022), “the global level of GHG emissions will largely depend on efficiency gains in India and other countries with high cattle populations and extensive production.” The biggest gains in inventory are predicted to occur in India, Pakistan, and Africa.

## The Important Role of Genetics in Sustainability

Genetic improvement is a particularly attractive approach to productivity enhancements, as following implementation genetic improvement is permanent and cumulative. One relatively simple technology that has been adopted by cattle producers in many high-income countries is artificial insemination (**AI**) to increase the intensity of selection by using genetically superior males. First introduced in the early 1940s (Foote, 2002), its use is widely adopted by the dairy industries in many countries. However, adoption has not been universal and is particularly low in extensive beef production systems where it is difficult to coordinate estrus synchronization protocols and perform AI on cows. The level of adoption of Brazil and India, the two countries with the largest cattle populations (Figure 1), has historically been low (<20%). However, recent efforts to increase adoption in both these countries have had some success.

In 1997, India became the largest milk-producing country in the world. In the period from 1951 to 2019, the country’s milk production increased over 1100%, whereas the increase in the bovid population was 24% and 153% for cattle and buffalo, respectively. This increased milk yield per cow was partially due to genetic improvement enabled by the adoption of AI (National Academy of Agricultural Sciences, 2020). The proportion of crossbred dairy cows increased from 17% in 1990–1991 to 38.3% in 2021–2022, and their share in the total cow milk production rose from 33.5% to 61.2% (Thakur and Birthal, 2023). Even so, the average annual milk production per adult female cow in India was only 1777 kg per animal in 2019 to 2020, compared to the global average of 2699 kg (Mitra et al., 2023), and the current U.S. average of almost 10,950 kg. The carbon footprint of milk is more than 2-fold higher on Indian dairy farms with annual milk yield less than 3,500 kg per cow, as compared to farms with greater milk yield (Mech et al., 2023). There is still high variation across states in daily milk yield, ranging from 1.49 to 13.31 kg/day for cows and 1.61 to 9.63 kg/day for buffalo suggesting considerable scope for increasing yields given locally available resources and technologies.

The use of X-chromosome-bearing “sexed” sorted semen in AI to increase the proportion of female calves born presents an intriguing opportunity in India (Thakur and Birthal, 2023). Given that cattle are revered in Hinduism, the culling or slaughter of infertile cows and male calves is prohibited, often resulting in these animals being abandoned on the streets or sent to gaushalas. Even absent the wide use of sexed semen, over the last three decades, the Indian cattle population has shifted significantly toward rearing more females (Thakur and Birthal, 2023). From 1992 to 2019, although Indian cattle numbers decreased by ~10 million head, the number of female cattle increased from 102.98 million to 145.91 million, while male cattle declined from 101.59 million to 47.6 million. This was achieved mainly through management, as X-sorted semen has only been available in India since 2017.

The Indian government has established AI stations that can produce sexed semen and is helping to encourage uptake by subsidizing the cost of the semen for farmers. Indian commercial dairy farmers’ willingness to pay for sexed semen was positively influenced by education level, herd size and attitude towards public extension systems (Verma et al., 2020). Increased uptake of AI and X-sorted sexed semen from genetically superior bulls in India and Pakistan to produce only female dairy cattle offers an approach to curtail the production of unwanted male calves in these large bovid populations. This would reduce the emission intensity of milk production without disrupting the livelihoods of dairy farmers in these two countries that are jointly on track to produce half of the projected increase in total milk production globally.

Brazil has the largest commercial cattle herd in the world at approximately 238 million head, with 43% dairy and 57% beef cattle. Interestingly, AI is more frequently used in the beef industry than the dairy industry in this country. Uptake in the beef industry has been very much driven by the development of protocols for timed artificial insemination (**TAI**) which allows a scheduled insemination of animals without the need to detect estrus. Using TAI increases both conception early in the breeding season and the genetic merit of resulting beef calves. Currently, 93.3% of inseminations in Brazil are performed using TAI (26.5 million TAI out of a total of 28.7 million doses of semen marketed). Furthermore, it is estimated that AI rates are approximately 25.9% in beef cattle and 12.0% for dairy cows and that the percentage of inseminated cows in Brazil will increase from the current 23% to 37% in the next 10 yr (Baruselli et al., 2019).

## Beef on Dairy: Genomic Selection and Sexed Semen

**Figure 3.**
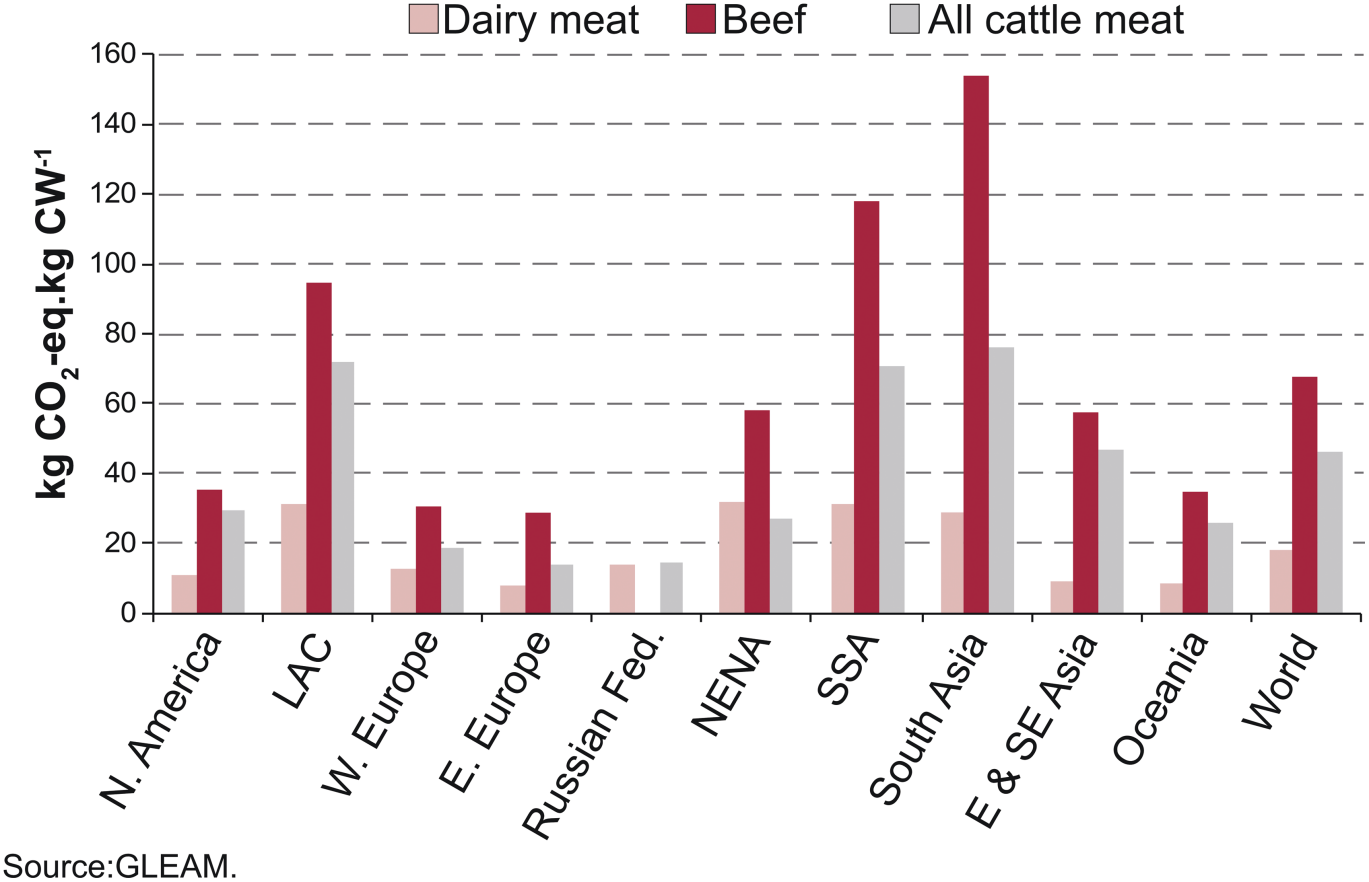
Regional comparison of emissions intensity per kilogram of carcass weight for animals originating from dairy versus specialized beef herds (Opio et al., 2013). LAC = Latin America and the Caribbean, NENA = Near East & North Africa, SSA = Sub-Saharan Africa. Reproduced from Opio et al., (2013) Greenhouse gas emissions from ruminant supply chains – A global life cycle assessment. Food and Agriculture Organization of the United Nations (FAO), Rome.

Although milk and beef are often seen as distinct foods, and in some countries, there are specialized beef and dairy production systems, the two are obviously interconnected. For example, in the European Union, 80% of beef comes from the dairy industry (Berry, 2021). Globally, 45% of beef comes from dairy production systems (Opio et al., 2013). On average, beef produced in specialized beef herds has almost four times the emissions intensity of beef from dairy farms which has historically been a byproduct of dairy production, comprised mainly of old cows at the end of their productive life (Figure 3).

The past decade has seen an explosion of genotyping and sequencing data that are currently being used to develop genomic breeding values. An evaluation of the impact of 7 yr of genomic selection in the U.S. dairy cattle shows that the rates of genetic gain per year increased 50% to 100% for high heritability traits, such as milk yield, and 300% to 400% for low heritability traits, such as somatic cell counts (**SCCs**, a measure of udder health) and daughter pregnancy rate (García-Ruiz et al., 2016). The combination of genomic selection to enable the identification of genetically superior dairy cows, combined with X-sorted sexed semen to produce female calves, has allowed producers to produce replacement heifer offspring from only genetically superior cows, typically young heifers. This has allowed the opportunity of using embryos or semen from beef breeds to artificially inseminate genetically inferior “low end” dairy cows thereby producing crossbred “Beef on Dairy” calves better suited to beef production than the male and surplus female dairy calves that historically left the dairy production system destined for veal, pet meat, or even euthanasia.

The National Association of Animal Breeders in the United States reported a 540% increase in domestic beef semen unit sales in the past decade from 1.67 million straws in 2012 to 9 million straws in 2022, indicating increased beef semen usage in dairy and beef breeding programs alike. It is almost 30 yr since the first use of sexed semen was reported and has been showing a double-digit growth in usage year on year. It is estimated in the United States that 20% of dairy heifers and 2% of cows are bred with sexed semen. Estimates indicate that sexed semen is rapidly approaching 30% of the total AI market share in North America (Seidel and DeJarnette, 2022). In 2023 in the United Kingdom, sexed semen made up 76.5% of all dairy semen sales, up from 70.5% in 2022 (Agriculture and Horticulture Development Board [AHDB], 2023). The British survey also showed that beef semen sales to the dairy herd had risen to 48%, up from 45.3% of total sales in 2021.

This is important because the production of dairy beef generates 18.4 CO_2-eq_ per kg CW as compared to 67.8 kg CO_2-eq_ per kg CW with production derived from specialized beef herds (Opio et al., 2013). This is partly because the diet of beef cows in extensive cow–calf operations is typically poor-quality roughage including leaves, grass, silage, and crop residues with low digestibility which increases dry matter intake and subsequently emissions, and additionally, because the emissions required to produce beef from dairy production systems are allocated in life cycle analysis between milk production and live weight sold for meat based on the relative energy requirements for the production of these coproducts, approximately 85% and 15%, respectively. Additionally, most of the emissions from specialized beef herds come from the breeding stock (i.e., cows) that produce no product other than weaning a calf, and these animals account for 55% to 97% of feed requirements and 52% to 97% of the methane emissions from beef produced in these systems. On average, beef from calves originating in dairy-based systems in developed countries showed 41% lower emissions (range 13% to 76%) as compared to calves derived from beef production systems (de Vries et al., 2015). Dairy beef increases the proportion of dairy farm income generated by the sale of animals, and these animals might also attract an additional premium in the future if a value-added market focused on climate-friendly beef products develops.

The positive economic return on the combined use of genomic selection, AI with sexed female dairy semen, and beef semen to produce dairy beef means this breeding strategy has been adopted rapidly by many dairy producers in many high-income countries with little public investment. The wide adoption of the relatively simple, proven innovations (AI, TAI, sexed semen, genomic selection) discussed in this paper thus far have had, and will continue to have, considerable potential to improve the efficiency of milk and beef production in both high-income and developing countries. The challenge in low-income countries is that these technologies are often too expensive or difficult to implement in the absence of the required infrastructure and technical expertise. The adoption of agricultural technology in now-developed countries has historically been done in collaboration with publicly funded extension efforts to work with developers to field test and refine products, optimize efficient and practical protocols, and ultimately promote the adoption of cost-effective, evidence-based agricultural innovations. Such a strategy will likely be required to facilitate the adoption of these proven technologies in low-income countries to more efficiently produce animal protein with improved environmental and economic sustainability.

## Future Uses of Genetic Improvement Technologies

One technology that has not been utilized in bovine genetic improvement programs to date is “genetic modification,” or more precisely genetic engineering (**GE**), and recently gene editing (GnEd). These terms refer to “modern biotechnologies” that are used to introduce intentional genomic alterations (**IGA**) in the genome of cattle to produce useful phenotype(s). Although there have been compelling examples of GE cattle for traits like disease resistance (Wall et al., 2005), the global pushback against the use of GE technologies in plants, combined with the uncertain and costly process associated with obtaining regulatory approval, has disincentivized global academic and investor interest in the commercialization of cattle produced using modern biotechnologies (Van Eenennaam et al., 2021).

GnEd offers a potential opportunity to alter that narrative. Due to the fact that the technology is so versatile, it opens up exciting opportunities ranging from the introgression of valuable single-gene traits like polled, heat tolerance, and disease resistance (Carlson et al., 2016; Rodriguez-Villamil et al., 2021; Workman et al., 2023) to novel applications like the generation of single-sex offspring, e.g., all female offspring (Kurtz et al., 2021). Furthermore, it also offers intriguing possibilities to alter breeding program design through surrogate sire (Gottardo et al., 2019) and in vitro breeding approaches (Goszczynski et al., 2018). The former presents an opportunity to reduce the genetic lag between the seedstock and commercial sector via the natural service delivery of elite genetics through surrogate sires carrying a germline derived from a genetically elite donor bull, and the latter offers the opportunity to dramatically reduce the generation interval of cattle through the development of gametes in cell culture, rather than sexually mature cattle. These developments open up new, hitherto unforeseen possibilities to accelerate the rate of genetic improvement of cattle further by incorporating genomic information, advanced reproductive technologies, and precision breeding methods into conventional breeding and selection programs (Mueller and Van Eenennaam, 2022).

**Figure 4.**
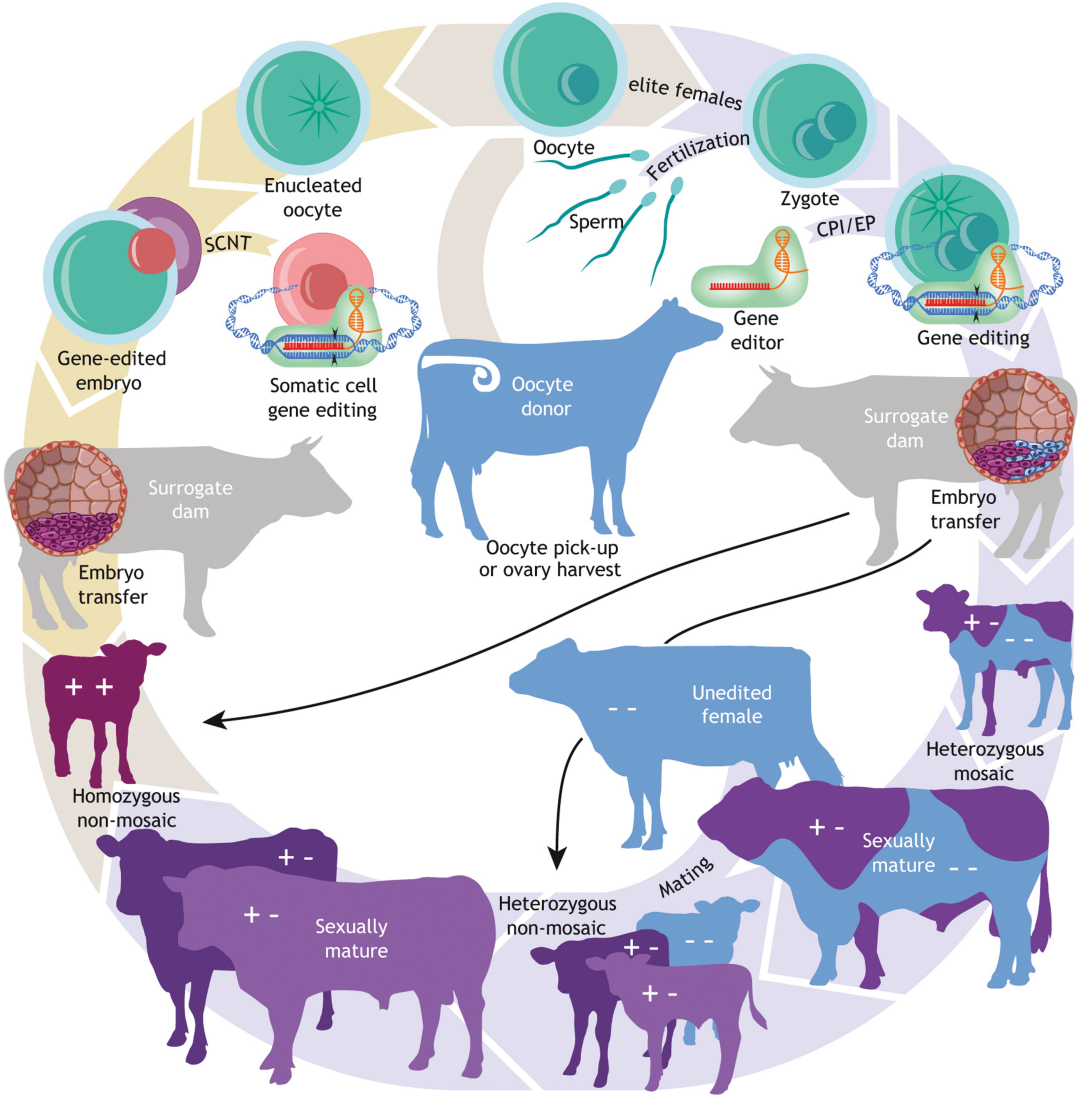
Steps for producing genome-edited mammalian livestock through somatic cell nuclear transfer (SCNT) cloning or zygote microinjection. Schematic showing the typical steps involved to produce homozygous, non-mosaic livestock by either SCNT cloning of screened genome edited somatic cells (yellow arrows) or cytoplasmic injection (CPI)/electroporation (EP) of one-cell zygotes (purple arrows) with genome editing components. Reproduced from Bishop and Van Eenennaam (2020) under a CC-BY license.

GnEd involves using a site-directed nuclease to introduce a double-strand break at a targeted location in the genome. It can be used to introduce targeted knock-outs (**KO**) of specific genes through nonhomologous DNA end joining or introduce the specific allelic variants (cisgenic) from the same species, and conceptually entire genes or transgenes as dictated by the homology-directed repair template nucleic acid sequence, rather than employing “artificial” selection, cross-breeding, and/or GE using random integration (Van Eenennaam, 2017). In mammals, GnEd can be performed in cell culture followed by somatic cell nuclear transfer (**SCNT**) cloning (Figure 4). To date, SCNT has been the primary method to deliver nuclease-mediated genetic changes into livestock (Tan et al., 2016). The advantage of SCNT is that the genome-edited cell line can be genotyped and/or screened prior to transfer into the enucleated oocyte to ensure that the desired edits, and no off-target edits, have occurred. The disadvantage is that there are well-documented drawbacks associated with cloning including embryo losses, postnatal death, and birth defects. Alternatively, editing can be undertaken in the developing embryo, in which case, the genotype of the animal will not be known until after it is born. One problem associated with direct editing of zygotes is mosaicism, the presence of more than one genotype in an animal.

To date, GnEd has been used on a limited scale to target traits in cattle including disease resistance, yield, welfare, and heat tolerance. It is worth noting that most complex traits are typically impacted by many different genes (i.e., polygenic or qualitative). It is unlikely that all of the genes impacting such traits are known nor is it typically evident which might be the desirable molecular edits for these genes (i.e., what is the sequence of the preferred allele). It is likely that editing will be focused on large effect loci and known targets to correct genetic defects or decrease disease susceptibility, and conventional selection powered by genomic selection will continue to make progress in selecting for all of the many small effect loci that impact the complex traits that contribute to the breeding objective. To become an important driver of genetic change, genome-editing methods must seamlessly integrate with conventional animal breeding programs. That means that editing methods must reliably produce germline-edited animals to be the next generation of parents, and these animals have to be able to contribute to the breeding scheme in a timely fashion.

Both KO and cisgenic applications do not introduce novel DNA and resemble genetic variations that can be found naturally in the genome. In many countries, GnEd animals that could have been achieved using conventional breeding are not being regulated or governed differently from animals produced using conventional breeding (Hallerman et al., 2024). GnEd can also be used to introduce DNA from another species by using a donor template with exogenous DNA sequences, in which case, the resulting animal carries foreign or “transgenic” DNA, and despite the known insertion site of the transgene, it is treated as a GE animal, also defined as a genetically modified organism (**GMO**) or living modified organism (LMO) from a regulatory perspective.

**Figure 5.**
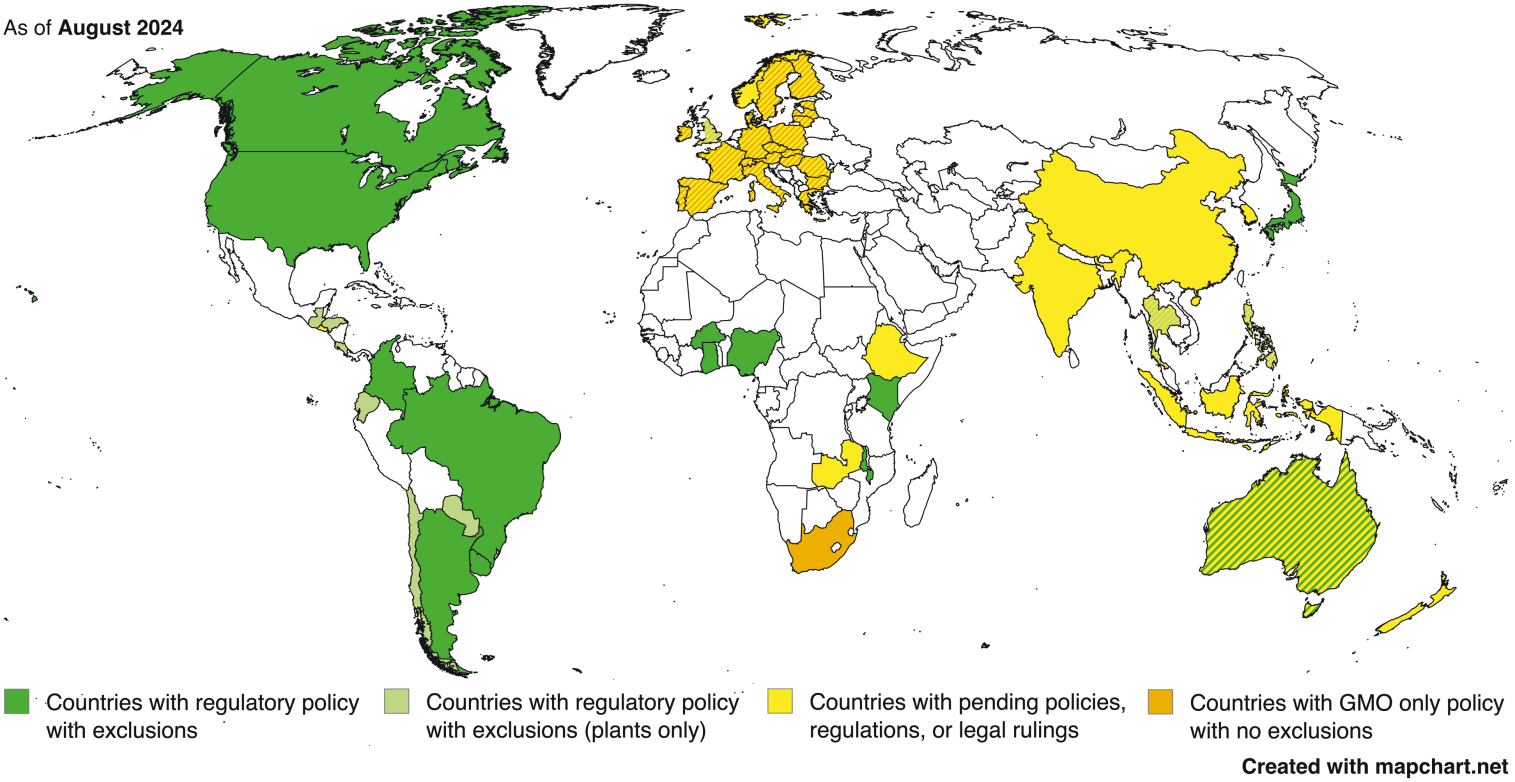
Countries that show progress in the development of policies for oversight of GnEd agricultural animals. Green indicates that products without a novel combination of DNA are excluded from GMO regulations. Image created based on published data (Wray-Cahen et al., 2024).

The GnEd animal applications that have undergone a regulatory process that allows some type of commercial sale of products are listed in Table 1. These authorizations of GnEd product sales are not necessarily traditional approvals in the sense of GE crop approvals for commercialization/planting and importation. Rather a determination has been made by the relevant regulatory authority in the listed country that these products are low risk (USA) or non-GMO (Argentina, Brazil, Colombia, and Japan) and can proceed to market. The regulatory picture for the products of GnEd animals varies by country and region based on the applicable laws and other forms of governance, and regulations within a country may even vary between the animal and plant kingdoms (Figure 5). It is notable that the countries that have regulatory policies that exempt low risk or non-GMO products produced through GnEd from mandatory premarket GE regulatory approval dominate Table 1.

**Table 1.** GnEd animal products that have undergone regulatory review in different countries

Country	Common name	Trait	Gene targeted	Year
Argentina	Nile Tilapia	Increased yield	Myostatin	2018
	Beef Cattle	Heat tolerance	Prolactin receptor	2020
	Dairy Cattle	Heat tolerance/polled	Prolactin receptor/P_c_ polled	2020
	Cattle	Increased yield	Myostatin	2021
	Other species	Undisclosed as not required for non-GMO products		
Brazil	Nile Tilapia	Increased yield	Myostatin	2019
	Beef Cattle	Heat tolerance	Prolactin receptor	2021
	Dairy Cattle	Heat tolerance	Prolactin receptor	2023
	Cattle	Increased yield	Myostatin	2021
	Pig	PRRS virus-resistance	CD-163	2024
Colombia	Pig	PRRS virus-resistance	CD-163	2023
Japan	Red Sea Bream	Increased yield	Myostatin	2021/2022
	Tiger Pufferfish	Faster growth	Leptin receptor	2022
	Olive Flounder	Faster growth	Leptin receptor	2023
USA	Beef cattle*	Heat tolerance	Prolactin receptor	2022

*Enforcement discretion was given for two specific beef cattle, not for all gene-edited (GnEd) cattle with this edit. If a “low risk” enforcement discretion decision is made, the developer is not expected to submit a drug approval application for the intentional genomic alteration (IGA).

Kyoto-based start-up Regional Fish Co., Ltd. started selling GnEd red sea bream and tiger pufferfish in Japan following a 2021 determination by regulatory authorities that these GnEd KO fish were “non-GMO” and presented no adverse effects to human health. In contrast to the GE experience, these applications were first reported in the scientific literature in 2018 and 2019, only a couple of years prior to their sale as food. Additionally, in 2023, a leptin receptor KO GnEd olive flounder with improved feed efficiency and growth rate, also developed by the Regional Fish Co. Ltd., was similarly allowed to proceed to market in Japan following the provision of molecular data and their determination that these fish were also “non-GMO.”

Interestingly, given previous experience with historical GE food controversies in Japan, it was anticipated that the introduction of GnEd fish into the food supply would be met with strong resistance from consumers. However, despite petitions being made by some consumer groups, they did not receive sustained attention and press coverage was mostly positive. The authors of a paper reporting on the reasons for this lack of a mass mobilization against GnEd fish in Japan suggested that this was partly due to the improved streamlined regulatory approaches that aimed to strike a balance between scientific discussion and public demand, the fact these fish were commercialized by the local academic professor rather than a large multinational company, and a “recent increase in public awareness of the SDGs (sustainable development goals) & expectations for food technology may have accelerated the acceptance of technologies that are presented in the context of reducing environmental loads & contributing to society (Matsuo and Tachikawa, 2022).”

Bruce and Bruce (2019) considered two examples of GnEd in livestock, hornless (polled) cattle and disease-resistant pigs, from the perspective of Responsible Research and Innovation. They suggested that the knowledge gap of the public regarding current practices in livestock agriculture could lead to unexpected outcomes from public consultations. For example, if an argument is made regarding GnEd to introduce the polled allele to the advantage of polled cattle, this might not be immediately obvious to those not versed in agricultural practice, and more generally, “the need for dehorning may be considered shocking by some publics” [9]. Applications for reduced antibiotic use, greenhouse gas emissions, and reduced possibility of transmitting zoonotic diseases were all deemed acceptable in a consultation performed by the UK Royal Society (van Mil et al., 2017). Although it should be noted that a major preoccupation of the participants in the United Kingdom was to ensure that genome editing was used to address inequality. The participants were particularly concerned about who owns the technology, who would benefit from its use, and whether it could be used to unfairly obtain monopoly power. This raises interesting questions regarding the fit-for-purpose of the expensive regulatory evaluation and approval approaches that have been proposed for GnEd animals in the US and EU; as such, a regulatory approach tends to advantage large companies and incentivize intellectual property protection.

## The Opportunity Cost of Inaction

Debates about the introduction of new breeding innovations are not new. When AI was first proposed, it also faced several obstacles. According to Foote (Foote, 2002), “The general public was against research that had anything to do with sex. Associated with this was the fear that AI would lead to abnormalities. Finally, it was difficult to secure funds to support research because influential cattle breeders opposed AI, believing that this would destroy their bull market.” Analogous arguments have been leveled against GE by the organic and natural food industry, alongside activist interests. Although the use of GE technology has been safely deployed in crop breeding and currently 12% of global cropland is planted with GE varieties, the general public remains highly skeptical of the technology and uncertain of its safety (Funk et al., 2015). The motive behind communication efforts by academic scientists to correct this safety perception (Kloor, 2015; Scheufele and Krause, 2019) and evidence documenting the fact that the billions of livestock eat almost exclusively GMO feed have suffered no ill effects (Van Eenennaam and Young, 2014) and have been drawn into question. GMO critics (Malkan, 2022) have not engaged in an objective discussion of the evidence base and scientific data but have rather focused on ad hominem attacks suggesting that scientists who are publicly communicating the safety data of GMO have undisclosed vested interests with the biotechnology industry and are involved in spreading propaganda (Folta, 2018).

Scientists who are subject matter experts willing to defend unpopular positions on politicized topics have frequently become the target of online attacks and harassment campaigns, often intended to silence minority or conflicting viewpoints (Lewandowsky and Bishop, 2016). Unfortunately, this has often proven effective at discouraging those who are unwilling to subject themselves and their reputation to such harms. This is particularly problematic when the topic is agricultural innovations because ignoring advice from experts and basing food production decisions on ideology rather than evidence has a long history of catastrophic outcomes including the Soviet famine in 1932 to 1933, the Great Leap Forward in 1960 to 1962, the “Holodomor,” and most recently, the 2022 collapse of the Sri Lankan economy (Wijerathna-Yapa et al., 2023).

There were several features that marked the development of GE crops which facilitated the creation of a well-funded and lucrative opportunity to promote opposition to these crops. One was the lengthy and expensive regulatory step uniquely associated with commercializing products developed using GE meaning only large multinational companies were able to bring products to the market. It is estimated that the cost of bringing a GE crop to market exceeds $100 million. The second was competing business and advocacy groups who were able to monetize fear around the method to extract value (rent seeking) and provide their value-added (i.e., more expensive) product that avoided the use of that breeding method (e.g., the non-GMO Project and Organic label). Importantly, there was some way to track/label products produced with or without that breeding method to enable value-added marketing (Van Eenennaam, 2024). Market research suggests that food labels that focus on one attribute (or lack thereof), such as GMOs, resonate with consumers. Currently, there are more than 3000 verified brands, representing over 50,000 products that are non-GMO Project verified and net more than $26 billion in annual sales in North America (Ryan et al., 2024). Groups opposed to GE have indicated they plan to frame GnEd as GMO2.0. It is worth noting that when GE product consumers wanted were allowed to reach the market, e.g., GE GloFish and leghemoglobin as an ingredient in the Impossible Burger, GE status did not dissuade interested customers from purchasing them. GloFish currently commands around 15% of the U.S. market share for aquarium fish sales, and Impossible Foods reported record sales of $460 million in 2022, outperforming the $418.9 million sales of non-GMO competitor Beyond Meat in the plant-based alternative category.

Foote (2002) contended that in the case of AI, “The careful field-tested research that accompanied AI soon proved to the agricultural community that the technology applied appropriately could identify superior production bulls free from lethal genes, would control venereal diseases, and did result in healthy calves. Thus, fear was overcome with positive facts. The extension service played an important role in distributing these facts.” It is not clear that “facts” are going to alter the conversation around the safety of GE food, as there has never been persuasive factual evidence of adverse health effects directly attributable to consumption of foods derived from GE crops. However, there are convincing data on the impact of forestalling or forgoing the adoption of safe breeding innovations (Biden et al., 2018; Van Eenennaam et al., 2021). Documenting the value of genetic improvement to sustainability goals and the opportunity cost of stalling the adoption of new breeding methods may be one way to address the goal conflicts that arise when precaution is given precedence over all other considerations.

Dramatic genetic improvement gains would have been forgone if AI, or more recently genomic selection, had preemptively been shelved because of negative perceptions of their unnaturalness, or precaution regarding hypothetical future risks. There have been some attempts to model the opportunity cost associated with not improving efficiencies in cattle production systems. The FAO (2018) reported that if there were no improvements in dairy cow efficiency from 2005 to 2015, the total GHG emissions from that sector would have increased by 38%. Similarly, from 1964 to 2014, California dairy systems reduced GHG emissions per kg of energy- and protein-corrected milk (ECM) by ~45.0%, water use intensity by ~88%, and land requirements for crop production by ~89% in 2014 compared with 1964 (Naranjo et al., 2020). More generally, Capper and Bauman (2013) review the important role that improved productivity has played in improving the environmental sustainability of ruminant production systems.

## Public Perception of Genetic Improvement

In Japan, it was argued that public awareness of the SDGs was part of the reason for public acceptance of GnEd fish in the food supply (Matsuo and Tachikawa, 2022). This suggests that highlighting the benefits of GnEd foods in metrics of relevance and interest to consumers may help with positively influencing acceptance (Strobbe et al., 2023). An interesting example is likely to play out in the real time as the first GnEd pig is brought to market. Genus plc, a UK-based publicly traded animal genetics company, plans to commercialize GnEd PRRS-virus resistant pig in Brazil, Canada, China, Colombia, Japan, Mexico, and the United States, but they are not currently planning to attempt an approval in the EU. Rather, the company will continue to provide conventional, PRRS-susceptible pigs to the European market where the annual costs of this disease exceeds $1.63 billion (Cigan and Knap, 2022). The net present value of the costs associated with continued PRRS virus infections in the EU from 2020 to 2049 was estimated to be USD$28.86 billion (Van Eenennaam et al., 2021). Additionally, genus has commissioned a life-cycle assessment to approximate the environmental footprint of pork production using their genetic lines, with and without the PRRS virus resistance.

One study found that over 75% of American consumers favor labeling of GnEd foods (Lindberg et al., 2023). However, the current Bioengineered Food Label Act in the United States, enacted by the federal government in 2016 and implemented in 2022, does not apply to KO or cisgenic GnEd products that do not contain foreign or “transgenic” DNA from another species. Similarly, in countries where GnEd animals that could have been achieved using conventional breeding are not being governed differently from animals produced using conventional breeding, there will be no obvious way to track/label GnEd animals and their products. Tracing GnEd food products may prove difficult as GnEd alterations can resemble or exactly mimic naturally occurring mutations (Strobbe et al., 2023). It may be that developers want to voluntarily promote their GnEd product with some associated sustainability claim (e.g., disease-resistant pigs), but tracking milk, meat, and eggs from GnEd animals in commodity handling systems will be difficult unless the entire supply chain avoids or includes products from GnEd animals. Something analogous happened with recombinant bovine somatotropin (**rBST**) which was approved for use in the United States in the Fall of 1993. Although food safety risks were alleged but never verified, marketers saw an opportunity to obtain a premium by producing a product that avoided the use of rBST and disallowed producers wanting to use rBST from selling products into their supply chain. Although this was associated with the known tradeoff of increasing the environmental footprint of a glass of milk (Capper et al., 2008), this tradeoff was rarely, if ever, made explicit to consumers.

At this stage, it is yet to be seen if GnEd will suffer the same fate as GE. It may be that as consumers become more aware of the tradeoffs and conflicts that exist among the economic, environmental, and social pillars of sustainability, discussions around GnEd will evolve beyond the dichotomous framing that accompanied GE crops, especially for GnEd livestock applications that benefit animal welfare and health. Producing GnEd disease-resistant animals, genetically polled cattle, and animals better able to cope with a warming environment moves beyond the simple traits of insect protection and herbicide tolerance that were targeted in the first generation of GE crops. Disease-resistant animals would seem to be a rare triple win for sustainability—better for the animal, farmer, and the environment.

## Conclusion

Genetic improvement has contributed to the reduced emission intensity of milk and beef in the developed world. This has traditionally involved conventional selection using a range of technologies to identify and disseminate genetics from high-genetic merit animals. Genetic improvement has not been as rapid in LMIC, home to 76% of the global cattle herd and 75% of the global ruminant GHG emissions. There are a range of reasons for this including the multiple roles beyond food production that cattle play in the livelihoods of livestock keepers in the global South and the difficulty of implementing some proven techniques (e.g., AI and genomic selection) in the absence of the required infrastructure. GnEd is an emerging technology that could be usefully employed to introduce targeted genetic variation into livestock breeding programs. However, the regulatory status of GnEd animals varies among countries, potentially complicating the trade of GnEd animals and their products. Furthermore, groups who are opposed to GE have indicated they plan to frame GnEd as GMO2.0. The fate of GE offers a cautionary tale of how disproportionate regulatory burdens on low-risk technologies and negative public perception can effectively preclude the development and adoption of beneficial genetic innovations. Delaying access to genetic improvement technologies that help to accelerate the rate of genetic gain and address intractable problems like animal disease is associated with a high opportunity cost of unrealized benefits.

## About the Author(s)

Alison Van Eenennaam is a Distinguished Professor of Cooperative Extension in Animal Genomics and Biotechnology in the Department of Animal Science at the University of California, Davis, in the United States. She received a Bachelor of Agricultural Science from the University of Melbourne, and both an MS in Animal Science, and a PhD in Genetics from UC Davis. She was employed as a Cooperative Extension Livestock and Dairy County Advisor, and a Development Scientist at Calgene, prior to commencing her current position. Her extension program focuses on the use of animal genomics and biotechnology in livestock production systems. She has a multifaceted research program that has included work on the uses of DNA information and biotechnologies in beef cattle production systems, and the development of genome editing approaches for cattle and sheep. Dr. Van Eenennaam is a past recipient of the ASAS Rockefeller Prentice Award in Animal Breeding and Genetics. **Corresponding author**: alvaneenennaam@ucdavis.edu
